# Examining the impact of a change in maternity leave policy in Canada on maternal mental health care visits to the physician

**DOI:** 10.1007/s00737-024-01448-y

**Published:** 2024-02-27

**Authors:** Marie-Pier Larose, Catherine Haeck, Pierre Lefebvre, Philip Merrigan

**Affiliations:** 1 INVEST Flagship Research Center, Department of Psychology and Speech-Language Pathology, University of Turku, Turku, Finland; 2 Département des sciences économiques, Université du Québec à Montréal, P.O. 8888, Box “A”, Montreal, QC Canada

**Keywords:** Maternity leave policy, Mental health, Healthcare system, Regression discontinuity

## Abstract

**Purpose:**

Maternity leave is a critical employee benefit that allows mothers to recover from the stress of pregnancy and childbirth and bond with their new baby. We aimed to examine the association between the extension of a maternity leave policy and maternal use of mental health services and prescription drugs in a universal public healthcare system.

**Methods:**

This study uses administrative medical records from 18,000 randomly selected women who gave birth three months before and after an extension of the maternity leave policy. More specifically, mothers who gave birth after January 1st 2001, were entitled to 50 weeks of paid maternity leave, while mothers who gave birth before that date were entitled to only 26 weeks of paid maternity leave. Medical records were analyzed over a seven-year period (i.e., from October 1998 to March 2006). We examined the number and costs of mothers’ medical visits for mental health care in the five years following delivery, as well as maternal use of prescribed medication for mental health problems.

**Results:**

We found that mothers with extended maternity leave had − 0.12 (95%CI=-0.21; -0.02) fewer medical visits than mothers without a more generous maternity leave and that the cost of mental health services was Can$5 less expensive per women. These differences were found specifically during the extended maternity leave period.

**Conclusions:**

The extra time away from work may help mothers to balance new family dynamics which may result in less demand on the healthcare system.

**Supplementary Information:**

The online version contains supplementary material available at 10.1007/s00737-024-01448-y.

## Introduction

The widespread increase in women’s labor force in the 1970 and 1980 s led most developed countries to adopt national maternity leave policies to support parents in their efforts to care for newborns while remaining attached to the labor market (Bütikofer et al. 2021; Heshmati et al. 2023; Nandi et al. 2018). In the European Union (EU), a minimum of two weeks of paid parental leave is mandatory, and there is a binding directive (i.e., a legislative act that sets a target for all EU countries) providing for at least 14 weeks of maternity leave with variable wage replacement coverage (European Parliament 2022; Staehelin et al. 2007). In the rest of the world, paid maternity leave is common practice, averaging 18 weeks and extending beyond 6 months in many high-income countries (Nandi et al. 2018). However, some countries, such as the United States, still lack a national policy regulating maternity leave, and there are significant disparities among states (Gault et al. 2014; Stack et al. 2019; Van Niel et al. 2020). Governments generally provide two types of support to parents of young children: job-protected leave and publicly provided financial assistance. These provisions and guidelines have led to heterogeneous parental leave policies among high-income countries (Bütikofer et al. 2021; Ray et al. 2009; Staehelin et al. 2007).

Several systematic reviews have examined the associations between parental and medical leave policies and socioeconomic outcomes as well as maternal and child health (Aitken et al. 2015; Andres et al. 2016; Bütikofer et al. 2021; Nandi et al. 2018; Staehelin et al. 2007). The findings can be summarized in three points. First, reforms increasing the duration of paid, job-protected parental leave have improved women’s economic and employment outcomes (Baker and Milligan 2008a; Gault et al. 2014; Nandi et al. 2018). Second, access to paid parental leave appears to reduce rates of infant mortality and improve early life outcomes (Aitken et al. 2015; Khanam et al. 2016; Nandi et al. 2018; Van Niel et al. 2020), maternal physical health (Aitken et al. 2015; Andres et al. 2016), and breastfeeding uptake and maintenance (Andres et al. 2016; Baker and Milligan 2008b; Staehelin et al. 2007; Van Niel et al. 2020). Finally, longer and more generous maternity leave policies (i.e., paid versus unpaid leave) are associated with improved maternal mental health, but the optimal length of maternity leave benefits still need to be examined (Bütikofer et al. 2021; Heshmati et al. 2023).

### Childbirth, maternal mental health and maternity leave policies

Approximately 10% of women will experience clinical levels of depression in the postpartum period (Howard et al. 2014). Many women seek pharmaceutical care or referral for psychotherapy or counseling from their physicians (Petersen et al. 2018; Brockington 2004). Postpartum depression may increase vulnerability to subsequent episodes of major depression and other psychiatric disorders (Rasmussen et al. 2017).

The presence, generosity and length of maternal leave policies may foster mental health in the short- and long-term (Avendano et al. 2015; Heshmati et al. 2023; Hewitt et al. 2017; Van Niel et al. 2020). For example, in a large prospective cross-sectional study in Australia, Whitehouse et al. (2013) reported that women who took more than 13 weeks of paid leave were less likely to experience psychological distress than mothers who did not receive paid maternal leave (Whitehouse et al. 2013). More recently, using data from an Australian national survey, Bilgrami (2019) reported a significant decrease in postnatal depression among women who benefited from the introduction of a national parental leave program. Also, Dagher et al. (2014) exploited a variation in the length of unpaid leave and found that an increase in the length of leave was associated with a decrease in depressive symptoms up to six months postpartum. Though, these studies have limitations such as the use of unrepresentative samples, the lack of long-term follow-up and only a small number of studies used a robust study design – like a natural experiment – to infer causality.

Yet, in recent years, some studies took advantage of changes in maternity leave policies to examine its association with maternal mental health using methods for causal inference. In Denmark, Beuchert et al. (2016) analyzed how a 32-day increase in maternity leave - over the 244 days already offered - was associated with mothers’ mental health. Using administrative data with an instrumental variable method coupled with a regression discontinuity (RD) design, they found no effects of the policy change on mothers’ likelihood of being hospitalized for depression or on mothers’ use of antidepressants (Beuchert et al. 2016). On the other hand, based on Norwegian data, Bütifoker et al. (2021) reported long-term benefits of introducing four months of paid maternity leave on women’s physical and self-reported mental health by the time mothers reach age 40. Although, based on data from an Austrian reform, Chuard (2023) reported that the extension of maternity leave coverage from 1.5 years to 2.5 years resulted in a deterioration in maternal mental health. Finally, Baker and Milligan (2008a, b) analyze this particular Canadian federal reform and found that extended maternity leave increased maternal time away from work, as well as breastfeeding initiation and duration, but reported no effect on self-report maternal depression.

These recent studies add considerable value to the literature, but they focus either on how introducing a maternity leave policy (Bütikofer et al. 2021), or extending an already lengthy maternity leave policies (i.e., more than 1 year) associate with maternal mental health (Beuchert et al. 2016; Chuard 2023), or used survey data which may be more prone to selection bias and social desirability (Baker and Milligan, 2008). In the present study, we propose to examine the association between the extension of maternity leave benefits from 6 months to 1 year using health registry data from a country offering universal healthcare. This insurance scheme will allow us to reach population representativity, to reduce selection bias into the study (Aitken, 2015), and to examine longitudinally the impact of the policy. Finally, given that many countries cap their benefits at about 6 months, this study may provide evidence on whether an extension in this specific time period could benefit maternal mental health.

### Objectives

We investigated the impact of extending maternity leave benefits on mothers’ mental health visits to the physicians, the cost associated with these visits and the cost of mother’s prescribed medications for mental health problems. We also examined whether these potential effects occurred at a specific time period during the first five years after delivery.

## Methods

### Study design

#### Socio-political context

In the province of Quebec (Canada), all permanent residents and citizens have access to free medical care through a universal health insurance. We rely on data from the *Régie de l’Assurance Maladie du Québec* (RAMQ) which records physician and hospitals invoices, including medical acts related to pregnancy and childbirth. The RAMQ data includes details of each service rendered to the patient and the corresponding fees. Data were completely anonymized, and the project was approved by the regulatory authorities – the RAMQ board and the *Commission d’accès à l’information du Québec*.

Prior to January 1^st,^ 2001, biological and adoptive parents were eligible to parental leave if they accumulated 700 h of insurable employment during the year preceding the birth of their child. Eligible parents (mothers or fathers) were entitled to ten consecutive weeks of paid parental benefits. Biological mothers were also entitled to an additional 15 weeks of maternity benefits, for a total of 25 weeks of insurable income.

Significant changes were implemented in January 2001 (see Table 1) for parents of children born or adopted after December 31st. Importantly, from January 1^st,^ 2001, basic parental leave package was increased from 10 to 35 weeks, providing biological mothers with a total of 50 weeks of paid maternity benefits.

**Table 1:** Evolution of the parental benefits in the province of Quebec (Canada)

	Parental benefits before the extension	Parental benefits after the extension
Coverage	Canadian new parents	Canadian new parents
Eligibility	700 h of “insurable employment” over 1 year	600 h of “insurable employment” over 1 year
Basic replacement rate	55%	55%
Low-income replacement rate	65%	80%
Maximum insurable earnings	Can$39,000 Max. of Can$412/week	Can$39,000 Max. of Can$412/week
Duration	15 weeks maternity 10 weeks parental	15 weeks maternity 35 weeks parental
Self-employed workers	Not covered	Not covered

### Study participants

Our datasets include information on 18,000 randomly selected women who gave birth between October 1st, 2000 and March 31st, 2001. The sample was chosen using a random number generator by the RAMQ. This period corresponds to the three months before and after the introduction of the changes in maternity leave benefits. The datasets include all physician-provided medical services over a seven-year period (two years before delivery and five years after) for each mother. Exactly 9,447 mothers were exposed to the new maternity leave policy, while the remaining 8,553 mothers did not have access to the more generous maternity leave package. Out of the 18,000 mothers, 2,295 were on social assistance and therefore not eligible to maternity leave benefits – yielding our final analytical sample to 15,705 participants. About a third of the mothers (35%) were aged between 25 and 29 years old, and only 2% of the sample were aged between 15 and 19 years old and older than 45 years of age. Also, about a quarter of the birth (26%) were registered in the largest metropolitan region of the province of Quebec (i.e., Montreal). Finally, the prevalence of cesarian delivery among mothers was similar for mothers before and after the change in maternity leave policy, 20.39% and 20.57%. More information on the socio-demographic characteristics of all the birth in the population according to birth registries, a table describing the socio-demographic characteristics of our analytical sample and the distribution of our outcomes are available in supplementary material (Table S1, S2 and S3).

### Populational representativeness

The 18,000 women in our sample represent approximately 55% of all women giving birth in the province between October 1st 2000 and March 31st 2001, according to the Quebec Birth Registry (Institut de la Statistique du Québec 2023). To ensure that pre- and post-reform mothers were not systematically different in other dimensions not available in the RAMQ data, we compared participants and newborn characteristics by month between October 2000 and March 2001 using data from the Quebec Birth Registry (i.e., socio-demographic and pregnancy-related outcomes) and found that they were very similar on both sides of the discontinuity point (see Table S1).

### Measures

#### Independent variable

The independent variable was the mother’s eligibility for the more generous maternity leave package. Eligibility was determined by the day of delivery, with mothers who gave birth before January 1st, 2001, not eligible, while mothers who gave birth on or after January 1st were eligible.

#### Outcome variables

Use of maternal mental health services was measured with three indicators from the RAMQ registry during pregnancy and the five years following delivery. First, we examined the number of visits to the physician for mental health care and the fee-for-service billing costs for these visits. To determine whether the reason for the visit was a physical or mental health issue, we rely on the RAMQ coding scheme for billing medical procedures. However, mothers may also be treated by psychologists for their mental health issues rather than by general practitioners or psychiatrists, but these costs are not reimbursed by the RAMQ and, therefore, are not available in the datasets.

Next, we examined the cost of prescription drugs for mental health problems for a subset of mothers covered by the Public Prescription Drug Insurance Plan (PPDIP) (*N* = 4,241 mothers). In Québec, since 1997, all citizens must have insurance for prescription drugs. Some mothers are covered by a private plan, which an employer may offer as an employee benefit. Our datasets contain purchasing data only for mothers covered by the public insurance plan. The PPDIP records provide the pharmacotherapeutic reason for the drug as well as the total cost of each prescription in the public plan. We compared the cost of medical visits for mental healthcare between women covered by the PPDIP and mothers with private insurance plans for the two-year period before pregnancy and did not find meaningful differences.

All the costs for the invoices to the RAMQ and the PPDIP were deflated by price indices (from Statistics Canada, base year 2002) of medical services and drug prices in Québec.

#### Covariates

We adjusted our analysis with maternal age, whether the delivery was performed on a weekday or during the weekend, and region of delivery (*N* = 15) as these covariates are recorded in the RAMQ registry and can influence access to mental healthcare services (see Table S2 in Supplementary material). We investigated whether the mode of delivery (i.e., vaginal vs. cesarian) differed between mothers before and after the reform and found no meaningful differences, and therefore did not adjust for this covariate in the main analysis. In sensitivity analysis, we included this covariate in the RDD, and found no meaningful differences with the main analysis (see Table S7).

In the model examining mental health services use during pregnancy, we used mothers’ healthcare costs (physical and mental health) for the two-year period prior to pregnancy as a proxy for lagged health problems. For regressions performed on postpartum data, we included maternal service use during pregnancy to account for lagged health effects. The same strategy was applied when we investigated the association between the policy and the cost of prescription drugs (i.e., cost of prescription drugs two-year before and during pregnancy).

### Statistical analysis

#### Is an extended maternity leave policy associated with maternal mental health service use?

We used a strict regression discontinuity (RD) approach to estimate the association between the length maternity leave benefits and the number of visits to the physician for mental health care, the costs associated with these visits, and the cost of medications prescribed for mental health problems. The RD strategy assumes that the assignment to treatment and control (i.e., exposed or not to the extended maternity leave) is random at the discontinuity point. See Appendix 1, Figure S1 and S2 for more information on RD.

Specifically, for each outcome, we fitted a local polynomial regression on the forcing variable (in our case, date of birth) on both sides of the discontinuity, and estimated the change at the discontinuity point, comparing the fitted value of the polynomials at that point. Standard errors were estimated using the bias corrected double bandwidth method of Calonico et al. (2017). To ensure the robustness of our results, we used two different analytical methods: (1) the “conventional” approach of Hahn et al. (2001) and Van Der Klaauw (2008), and (2) the “bias-corrected” approach of Calonico et al. (2014).

#### Do these associations vary by a specific postpartum period?

To determine whether the extension of the maternity leave policy led to changes during a specific time period, we repeated the analysis defined above, restricting the outcomes to a specific postpartum period: (1) during pregnancy (nine months before the delivery date), (2) during the first six months post-delivery, (3) between six months and one-year post-delivery (4) between one and two years post-delivery, and (5) between two and five years post-delivery.

#### Sensitivity analysis

To investigate the robustness of our estimates, we restricted the sample to participants who were in a very short window before and after the discontinuity point (i.e., mothers who gave birth in the week before and the week after the introduction of the new maternity leave policy). We also reran our analysis while removing mothers who were at the 99th percentile of the distribution of our outcomes to ensure that extreme values did not exert a disproportionate effect.

## Results

### Participants

Descriptive statistics on the number of medical visits for mental health care and their associated costs, as well as the cost of prescription drugs are presented in Table 2. The average number of visits over pregnancy and the five-year postpartum period is 1.78 and the cost per visit averages Can$44.68 (US$33.39). Finally, on average, the cost of prescription drugs for mental health problems over the five years post-delivery was Can$108.11 (US$80.78).

**Table 2:** Descriptive statistics on maternal use of mental health care services and associated costs from pregnancy to 60 months post-delivery

Pre- and Post-natal Periods	Number of Visits for MH care			Cost for MH care (Can$)			Cost of Prescription Drugs for MH (Can$)		
		Mean (SD)			Mean (SD)			Mean (SD)	
	Pre-reform (*N* = 7,410)	Post-reform (*N* = 8,295)	All (*N* = 15,705)	Pre-reform (*N* = 7,410)	Post-reform (*N* = 8,295)	All (*N* = 15,705)	Pre-reform (*N* = 2,066)	Post-reform (*N* = 2,175)	All (*N* = 4,241)
Pregnancy until delivery (9 months)	0.12	0.11	0.12	5.89	5.17	5.51	3.39	3.00	3.19
	(0.93)	(0.79)	(0.86)	(59.49)	(42.08)	(51.04)	(39.62)	(26.44)	(33.51)
Delivery to six months	0.14	0.12	0.13	6.33	5.22	5.75	5.22	5.88	5.56
	(1.17)	(0.9)	(1.04)	(63.79)	(38.44)	(51.96)	(28.35)	(32.2)	(30.85)
Six to 12 months post-delivery	0.15	0.12	0.14	6.75	5.67	6.18	7.12	7.62	7.37
	(0.88)	(0.76)	(0.82)	(44.89)	(41.43)	(43.1)	(38.34)	(42.46)	(40.5)
12 to 24 months post-delivery	0.33	0.29	0.31	14.52	13.08	13.76	20	17.35	18.47
	(1.61)	(1.33)	(1.47)	(78.69)	(69.89)	(74.17)	(110.58)	(77.35)	(95.0)
24 to 60 months post-delivery	1.25	1.16	1.2	55.33	52.5	53.83	84.81	69.00	76.71
	(4.81)	(3.73)	(4.27)	(233.68)	(191.92)	(212.65)	(546.47)	(391.26)	(473.28)
Delivery to 60 months	1.87	1.69	1.78	82.94	76.48	79.53	117.16	99.51	108.11
	(6.43)	(5.02)	(5.73)	(318.62)	(259.17)	(288.76)	(629.46)	(475.54)	(555.87)

*Notes* MH = Mental Health; SD = Standard Deviation. Mothers on welfare are excluded from this table and subsequent analysis because they are not eligible to maternity leave benefits

### Number of mental health care visits and associated costs

**Fig. 1:**
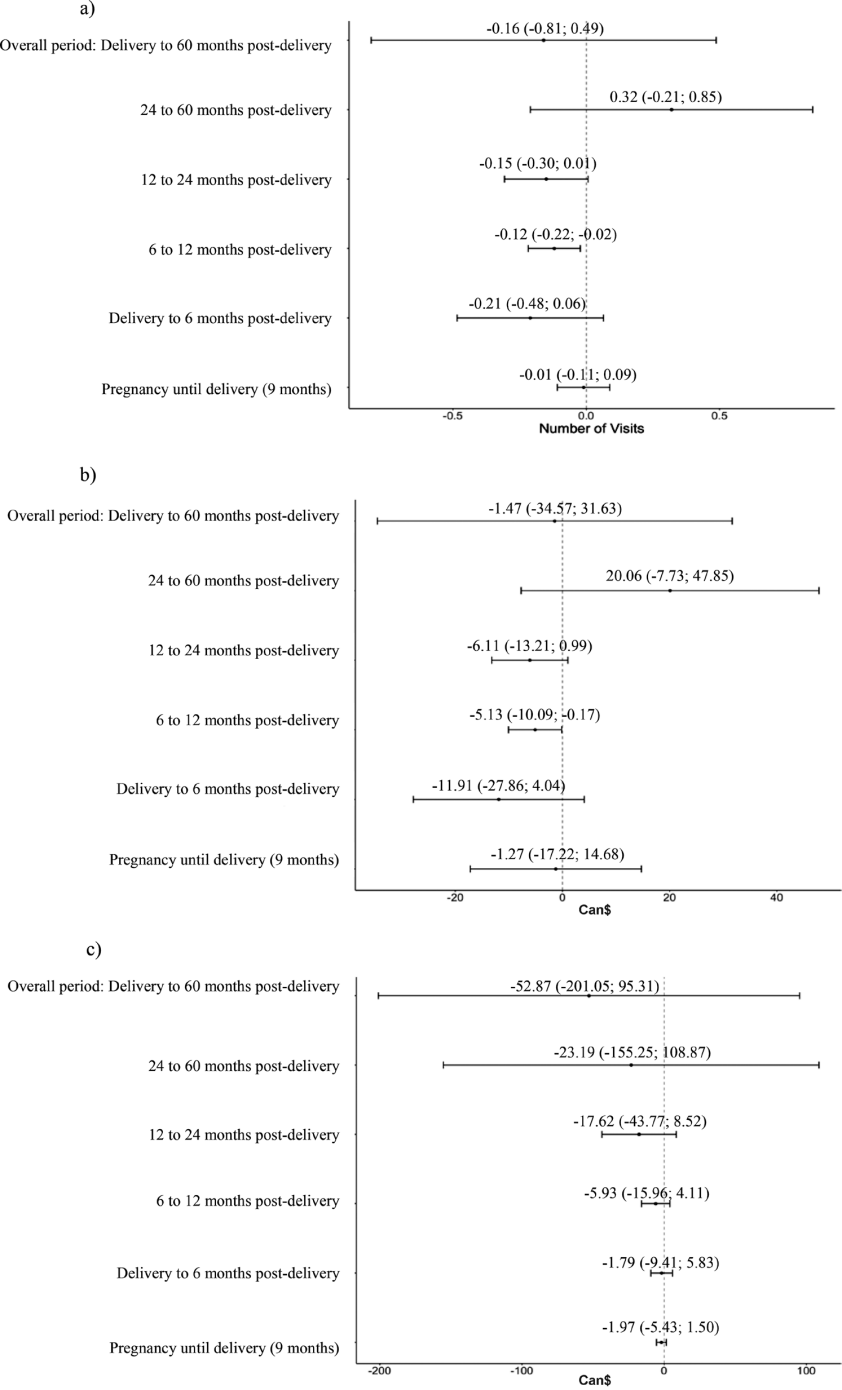
Regression discontinuity coefficients for **(a)** number of medical visits for mental health care, **(b)** cost of visit for mental health care, an **(c)** cost of prescriptions drugs. *Notes. *Regression coefficients per sub-period are displayed above their respective estimation marker with their 95% confidence intervals. The regression coefficients are drawn from the analysis using the bias-corrected robust method by Calonico et al. (2014). Double optimal bandwidth was estimated using following Calonico, Cattaneo, Russell, and Titiunik (2016) computational method

Figure 1 displays the RD regression estimates for the total five-year postpartum period as well as the estimates for each subperiod using to the bias-corrected methods (for RD charts, see supplementary Figure S3). Coefficients for the conventional method are presented in Table S4 in Supplementary material. The estimates represent differences in the number of medical visits for mental health care as well as differences in Can$ between women who received extended maternity leave and those who did not.

Interestingly, we found a negative association (i.e., a decrease in cost) during the 6-month to 12-month postpartum period for participants exposed to the extended maternity leave, which corresponds to the extended maternity leave period. Specifically, women exposed to the longer maternity leave required fewer visits for mental health issues (*B* = -0.12, *SE* = 0.05; representing a decrease of 6.41% on the average number of visits from delivery to 5 years post-partum) and the costs of these visits were lower (Can$5 less expensive per women; representing a decrease of 6.03% on the average cost from delivery to 5 years post-partum) during the specific six-month to 12-month postpartum period (see Fig. 1a and b).

### Prescription drugs costs

We found no association between the extension of the maternity leave policy and the use of prescription drugs for mental health problems, regardless of the time period studied.

### Sensitivity analysis

**Fig. 2:**
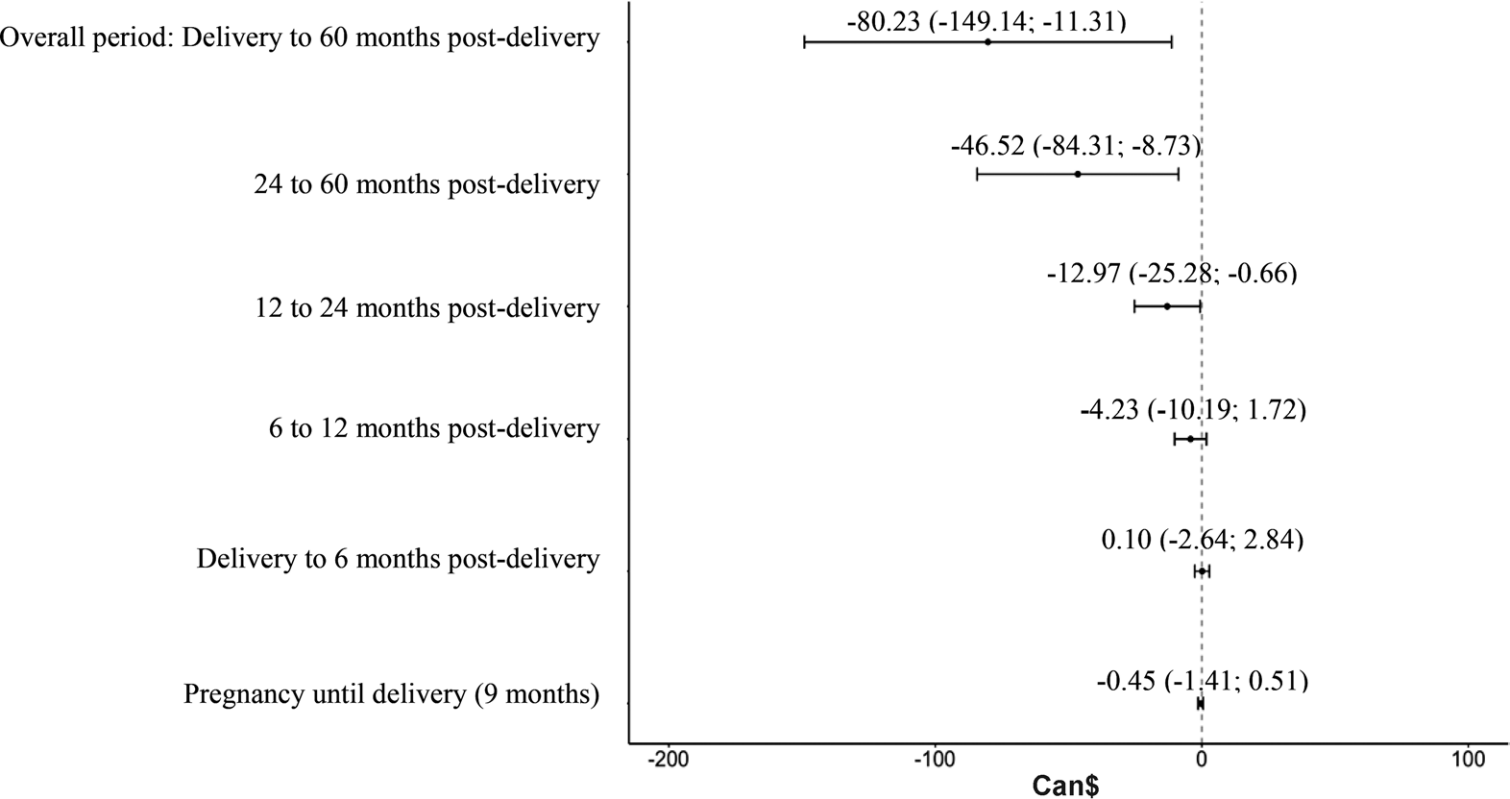
Regression discontinuity coefficients for cost of prescription drug for mental health problems, 99th percentile removed. *Notes.* Regression coefficients per sub-period are displayed above their respective estimation marker with their 95% confidence intervals. The regression coefficients are drawn from the analysis using the bias-corrected robust method by Calonico et al. (2014). Double optimal bandwidth was estimated using following Calonico, Cattaneo, Russell, and Titiunik (2016) computational method

Our estimates are rather precise, and our results on the number of mental health visits and the costs associated with these visits are robust to (a) restricting the sample to mothers who delivered around the discontinuity point as well as (b) removing the participants over the 99th percentile on our outcomes (See table S5 and S6 in Supplementary Material). Interestingly, once the extreme values were removed from the analysis (see Fig. 2), we found a significant overall decrease of Can$80.23 (US$60.24) in the cost of prescribed medications for mental health problems among participants eligible for the more generous maternity leave program.

## Discussion

Using a natural experiment study design paired with a RD approach, we found a small decrease (around 6%) in the number of visits for mental health care and their associated costs for mothers with more generous maternity leave. This diminution was specifically observed during the extended leave period (i.e., six to 12 months postpartum). Prior to the reform, most mothers would have returned to work during that period. Of interest, the decrease in costs during the six to 12 months postpartum was not followed by an increase in the 12 to 24 months period – when these mothers returned to work. This suggests that there might be a more optimal time for mothers to return the workforce. The six to 12 months post-delivery is a sensitive period of child development, when mothers are encouraged to continue breastfeeding (CDC, 2022) and infant sleep patterns are still developing (Hysing et al. 2014). The pressure of re-entering the workforce while continuing to breastfeed and having interrupted nights may take a greater toll on mother’s mental health, which may be less potent when the mother re-enters the workforce at 12 months postpartum.

The extension of the maternity policy has previously been linked with an increase in the duration of breastfeeding (Baker & Milligan, 2008), therefore, the impact of the policy on maternal mental health could be partially explained by the positive association between breastfeeding and maternal mental health (Yuen et al. 2022), and this mechanism deserves further investigation. Furthermore, our results suggest that the maternity leave may have had an impact on mothers who experience depressive symptomatology in the post-partum period (i.e., up to one year), but the impact might not extend for women who may experience more chronic mental health problem.

Our study was not designed to assess the cost-effectiveness of maternity leave policies, but to leverage registry data to examine how extending maternity leave benefits associates with maternal mental health during a crucial postpartum period (i.e., 6 to 12 months), while using a robust analytic approach to infer causality. By reporting a distal association between maternity leave benefits and maternal use of medical services for mental health care, we suggest that an even larger association may be observed on proximal maternal and familial outcomes. Future studies should also explore the potential moderation effect of lifelong mental and physical health as well as socio-economic status on the impact of the change in maternity leave policy on maternal health outcomes.

In addition, the results must be put into perspective with other significant determinants of health. It is interesting to note that this study was conducted in the province of Québec (Canada), where all women and children have access to universal health care, and after the introduction of a universal childcare reform (Lefebvre and Merrigan 2008). Maternity leave benefits are just one government tool to potentially improve mothers’ mental health. However, maternity leave policies face the challenge of improving maternal and child health outcomes while avoiding widening the gender gap in human capital, as women may lose some of their human capital by temporarily leaving the labour market and may have difficulties re-entering the labour force at the end of their maternity leave (Kalb 2018) - which can also harm their long-term mental health.

The first strength of our study is its strong design and analytical approach to inferring causal associations. Second, we performed our analysis on a representative and large sample of mothers covered by the same universal public healthcare system. Third, although we were not able to identify mental health reasons (e.g., depression, anxiety, substance use problems) for recorded medical visits, we relied on objective measures of mental health based on the services provided by physicians and the diagnoses associated with those services. Finally, the longitudinal administrative data allowed us to distinguish between the short- and long-run associations between the extension of a maternity leave policy and maternal mental health and to examine this association during the extended period.

The first limitation of this study is that psychological services in the private sector are not reimbursed by RAMQ and are therefore absent from the analysis. Our results potentially underestimate the actual association between the change of policy and mothers’ use of mental health services. Second, our data on prescription drug was more sensitive to outliers and limited by the restricted number of mothers on the public plan who may not have experienced severe enough symptomatology to rely on the use of prescription drugs. Third, although we used an RD approach, we are not immune to the potential impact of undocumented confounders that could bias our RD estimates. Fourth, we did not distinguish between health care use and health care expenditure, which could have provided additional information on the impact of the change in policy (Deb and Norton 2018).

## Conclusions

Mothers may benefit from longer parental leave, but this association does not extend after they rejoin the labour force. In addition, the COVID-19 pandemic has altered work environments, where full or partial remote work is more common (Fan and Moen 2022). Studies on the transition from maternity leave to work life in this new era are needed to guide future work-family balance policies.

## Electronic supplementary material

Below is the link to the electronic supplementary material.

Supplementary Material 1

## Data Availability

CH and PM had full access to all the data in the study and takes responsibility for the integrity of the data and the accuracy of the data analysis. No plan on data sharing.
