# Supplementary material for: Examining the impact of a change in maternity leave policy in Canada on maternal mental health care visits to the physician
Source: Arch Womens Ment Health. 2024 Feb 27;27(5):775–83. doi: 10.1007/s00737-024-01448-y (PMC11405480; doi:10.1007/s00737-024-01448-y)
Supplement: Supplementary file 1 — Supplementary Material 1 [file 737_2024_1448_MOESM1_ESM.docx]

**Supplementary Material**

[Appendix 1: Regression Discontinuity Approach 2](#_Toc158973904)

[Table S1: Characteristics of mothers and newborns by selected year and month 5](#_Toc158973905)

[Table S2: Maternal age, region of residence and mode of delivery 6](#_Toc158973906)

[Table S3. Distribution of maternal use of mental health services and associated costs from pregnancy to 60 months post-delivery. 7](#_Toc158973907)

[Table S4. Non-parametric regression discontinuity coefficients for number of visits, costs of mental health care and cost of prescription drug for mental health problems using the conventional method. 8](#_Toc158973908)

[Table S5. Non-parametric regression discontinuity impacts for number of visits, costs of mental health care and cost of prescription drug for mental health problems, restrained sample near the discontinuity point. 9](#_Toc158973909)

[Table S6. Non-parametric regression discontinuity impacts for number of visits, costs of mental health care and cost of prescription drug for mental health problems, 99th percentile removed 10](#_Toc158973910)

[Table S7. Non-parametric regression discontinuity coefficients for number of visits, costs of mental health care and cost of prescription drug for mental health problems using the robust method and adjusting for type of delivery (cesarian vs vaginal). 12](#_Toc158973911)

[Figure S1. Birth pattern in the one month prior and after the change of maternity leave policy 14](#_Toc158973912)

[Figure S2. Running variable density test 15](#_Toc158973913)

[Figure S3: RDD chart of mental health costs by periods around the discontinuity point. 16](#_Toc158973914)

# **Appendix 1: Regression Discontinuity Approach**

We estimated the impact of a change in maternity leave package on the cost of maternal mental health on the healthcare system using a strict regression discontinuity approach (RD) following two analytical methods: (1) the “conventional” approach of Hahn et al ^1^ and Van Der Klaauw ^2^, and (2) the “bias-corrected” approach of Calonico et al. ^3^.

The RD strategy assumes that the assignment to treatment and control (i.e., exposed or not to the extended maternity leave) is random at the discontinuity point. More specifically, to be unbiased, the RD strategy requires that mothers do not time births in response to the reform. If they do, it is possible that the treatment group (mothers giving birth after December 31^st^, 2000) and the control group (mothers giving birth before January 1^st^, 2001) differ in terms of unobservable health factors even if they share the same distribution of observed characteristics. Manipulation may occur if mothers are aware that the reform will soon be implemented. In practice, it is unlikely that mothers time their pregnancies as the legislative process was long, the exact date the new policy would be introduced was not mentioned prior to the end of the legislative process and, furthermore, timing of conception and date of birth cannot be completely controlled by parents as we get closer to the discontinuity point.

More specifically, we fitted a local polynomial regression of outcomes on the forcing variable (in our case, date of birth) on both sides of the discontinuity, and estimate the causal effect at the discontinuity point, by comparing the fitted value of the polynomials at this point. The moment when the policy was introduced clearly divides mothers who were and were not exposed to the new extended maternity leave benefits.

Standard errors are estimated using the bias corrected double bandwidth method of Calonico et al. ^4^. The use of bandwidth is a strategy based on local randomization and limits the analysis to observations that lie within the close area of the discontinuity point where the functional form is more likely to be linear. The main challenge is selecting the right bandwidth. Our optimal bandwidth varies from approximately 20 to 30 days in most regressions.

**Reassuring that there is no manipulation of the timing of birth.**

To ensure that mothers did not time their time of birth in response to the change in policy like it has been previously reported in relation to maternity benefits (see Gans and Leigh, 2009), we conducted a visual appreciation of the number of births during the month prior and after the change in maternity leave benefits. Figure S1 presents the number of mothers delivering by day during the month prior and after the change in maternity leave policy. We then perform the McCrary (2008) test that seeks to identify jumps in the density of the number of births at the discontinuity. Figure S2 displays the estimated density of the running variable. There is an apparent break at the discontinuity, but we cannot reject the null hypothesis of no manipulation with the McCrary statistic. However, we estimate the effect of the policy change for several cases excluding days close to the discontinuity and come to the same conclusions.

**Investigating changes in demographics and child’s birth outcomes after the introduction of the policy.**

To validate that mothers before and after the reform are not systematically different in other dimensions not available in the RAMQ data, we also use information from the administrative birth registers. These registers contain all births in Quebec. Table S1 present mothers and newborn mean characteristics by month, between October 2000 (month 10) and March 2001 (month 3). The statistics indicate that averages of variables such as the mothers’ place of birth, age, mother tongue, family status, levels of education, sex of the child, birth weight, probability of low birth weight (below 2500 grams), mean number of gestation weeks, birth order and single birth, are very similar on both sides of the discontinuity.

**References**

1. Hahn J, Todd P, Van der Klaauw W. Identification and Estimation of Treatment Effects with a Regression-Discontinuity Design. *Econometrica*. 2001;69(1):201-209.

2. Van Der Klaauw W. Regression–Discontinuity Analysis: A Survey of Recent Developments in Economics. *LABOUR*. 2008;22(2):219-245. doi:10.1111/j.1467-9914.2008.00419.x

3. Calonico S, Cattaneo MD, Titiunik R. Robust Nonparametric Confidence Intervals for Regression-Discontinuity Designs. *Econometrica*. 2014;82(6):2295-2326. doi:10.3982/ECTA11757

4. Calonico S, Cattaneo MD, Farrell MH, Titiunik R. Rdrobust: Software for Regression-discontinuity Designs. *Stata J*. 2017;17(2):372-404. doi:10.1177/1536867X1701700208

5. Gans, J. S., & Leigh, A. (2009). Born on the first of July: An (un) natural experiment in birth timing. *Journal of public Economics*, *93*(1-2), 246-263.

# **Table S1: Characteristics of mothers and newborns by selected year and month**

| **Birth month** | | **Oct 2000** | | **Nov 2000** | | **Dec 2000** | | **Jan 2001** | **Feb 2001** | | **Mar 2001** |  |  |
| --- | --- | --- | --- | --- | --- | --- | --- | --- | --- | --- | --- | --- | --- |
| **Mother's Place of birth** | | | | | |  | | |  | | |  |  |
| Québec | | | 0.76 | 0.77 | | 0.77 | | 0.77 | 0.78 | | 0.79 |  |  |
| Rest of Canada | | | 0.05 | 0.06 | | 0.05 | | 0.06 | 0.05 | | 0.06 |  |  |
| Other | | | 0.19 | 0.16 | | 0.17 | | 0.16 | 0.16 | | 0.14 |  |  |
| **Age group of the mother at childbirth** | | | | | |  | | |  | | |  |  |
| Less than 17 | | | 0.00 | 0.00 | | 0.00 | | 0.00 | 0.00 | | 0.00 |  |  |
| 17-35 | | | 0.90 | 0.88 | | 0.89 | | 0.89 | 0.90 | | 0.90 |  |  |
| 36 or more | | | 0.10 | 0.12 | | 0.11 | | 0.11 | 0.10 | | 0.10 |  |  |
| **Mean age of the mother at childbirth** | | | | | |  | | |  | | |  |  |
| Age | | | 28.3 | 28.5 | | 28.3 | | 28.4 | 28.4 | | 28.6 |  |  |
| **Mother's mother tongue** | | | | | |  | | |  | | |  |  |
| French | | | 0.79 | 0.80 | | 0.80 | | 0.80 | 0.81 | | 0.81 |  |  |
| English | | | 0.12 | 0.11 | | 0.11 | | 0.11 | 0.11 | | 0.11 |  |  |
| Other | | | 0.00 | 0.01 | | 0.01 | | 0.01 | 0.01 | | 0.01 |  |  |
| **Family status of the mother** | | | | | |  | | |  | | |  |  |
| Couple | | | 0.88 | 0.86 | | 0.87 | | 0.87 | 0.85 | | 0.86 |  |  |
| Single parent | | | 0.07 | 0.08 | | 0.08 | | 0.08 | 0.08 | | 0.08 |  |  |
| **Mother's level of education** | | | | | |  | | |  | | |  |  |
| No diploma | | | 0.06 | 0.07 | | 0.08 | | 0.07 | 0.07 | | 0.07 |  |  |
| High school | | | 0.25 | 0.25 | | 0.25 | | 0.24 | 0.22 | | 0.22 |  |  |
| College | | | 0.29 | 0.29 | | 0.28 | | 0.30 | 0.31 | | 0.29 |  |  |
| University or more | | | 0.25 | 0.26 | | 0.25 | | 0.27 | 0.28 | | 0.31 |  |  |
| **Sex of child** |  | | | |  | |  | | |  | |  |  |
| Boy | | | 0.51 | 0.52 | | 0.52 | | 0.51 | 0.52 | | 0.51 |  |  |
| **Birth weight of child** | | | | | | |  | | |  | |  |  |
| Weight (grams) | | | 3373 | 3377 | | 3358 | | 3352 | 3358 | | 3362 |  |  |
| <2 500 grams | | | 0.04 | 0.05 | | 0.05 | | 0.05 | 0.05 | | 0.05 |  |  |
| =>2 500 grams | | | 0.95 | 0.94 | | 0.94 | | 0.94 | 0.94 | | 0.94 |  |  |
| **Gestation** | | | | | |  | | |  | | |  |  |
| Gestation (weeks) | | | 38.8 | 38.8 | | 38.8 | | 38.8 | 38.8 | | 38.8 |  |  |
| Less than 29 weeks | | | 0.01 | 0.01 | | 0.01 | | 0.01 | 0.01 | | 0.00 |  |  |
| 30-35 weeks | | | 0.04 | 0.04 | | 0.04 | | 0.04 | 0.04 | | 0.04 |  |  |
| 37 or more weeks | | | 0.92 | 0.93 | | 0.91 | | 0.91 | 0.92 | | 0.92 |  |  |
| **Birth order of the child and single birth** | | | | | | | | |  | | |  |  |
| 1 | | | 0.47 | 0.48 | | 0.48 | | 0.47 | 0.45 | | 0.46 |  |  |
| 2 | | | 0.35 | 0.34 | | 0.34 | | 0.36 | 0.38 | | 0.37 |  |  |
| 3 or more | | | 0.14 | 0.15 | | 0.15 | | 0.14 | 0.142 | | 0.14 |  |  |
| Single birth | | | 0.98 | 0.97 | | 0.97 | | 0.97 | 0.97 | | 0.97 |  |  |

*Note*s: The total of percentage age may not sum to 100% because missing observations are excluded.

# **Table S2: Maternal age, region of residence and mode of delivery**

| **Covariates** | **Oct. 2000-Mar. 2001**  Prevalence (SD) | Dec. 25-31 2000 | Jan. 1-7 2001 |
| --- | --- | --- | --- |
| **Maternal age** |  |  |  |
| 15-19 year olds | 0.02 (0.14) | 0.02 (0.16) | 0.03 (0.17) |
| 20-24 year olds | 0.19 (0.39) | 0.19 (0.40) | 0.20 (0.40) |
| 25-29 year olds | 0.35 (0.48) | 0.35 (0.48) | 0.35 (0.48) |
| 30-34 year olds | 0.29 (0.45) | 0.29 (0.45) | 0.27 (0.44) |
| 35-40 year olds | 0.13 (0.34) | 0.12 (0.33) | 0.13 (0.34) |
| 40-49 year olds | 0.02 (0.15) | 0.02 (0.15) | 0.03 (0.17) |
| **Region of residence** |  |  |  |
| 1 Bas-Saint-Laurent | 0.03 (0.16) | 0.04 (0.18) | 0.03 (0.16) |
| 2 Saguenay-Lac-Saint-Jean | 0.04 (0.19) | 0.04 (0.20) | 0.04 (0.20) |
| 3 Québec | 0.08 (0.27) | 0.10 (0.3) | 0.09 (0.28) |
| 4 Mauricie et Centre-du-Québec | 0.06 (0.23) | 0.08 (0.25) | 0.05 (0.21) |
| 5 Estrie | 0.04 (0.19) | 0.03 (0.18) | 0.04 (0.18) |
| 6 Montréal | 0.26 (0.44) | 0.24 (0.43) | 0.23 (0.44) |
| 7 Outaouais | 0.04 (0.20) | 0.04 (0.18) | 0.05 (0.22) |
| 8 Abitibi-Témiscamingue | 0.02 (0.14) | 0.02 (0.13) | 0.02 (0.13) |
| 9 Côte-Nord | 0.01 (0.11) | 0.01 (0.09) | 0.01 (0.12) |
| 10 Gaspésie-Îles-de-la-Madeleine | 0.01 (0.10) | 0.01 (0.11) | 0.01 (0.11) |
| 11 Chaudière-Appalaches | 0.06 (0.23) | 0.06 (0.23) | 0.05 (0.21) |
| 12 Laval | 0.05 (0.22) | 0.04 (0.20) | 0.04 (0.20) |
| 13 Lanaudière | 0.05 (0.22) | 0.06 (0.24) | 0.05 (0.23) |
| 14 Laurentides | 0.07 (0.25) | 0.06 (0.23) | 0.08 (0.27) |
| 15 Montérégie | 0.18 (0.38) | 0.18 (0.38) | 0.16 (0.38) |
| **Mode of delivery**  Cesarian | 0.20 (0.40) | 491  89 | 576  107 |
| Observations | 15,705 | 491 | 576 |

# **Table S3. Distribution of maternal use of mental health services and associated costs from pregnancy to 60 months post-delivery.**

|  | **Number of Visits for MH care** | | | | |
| --- | --- | --- | --- | --- | --- |
| **Pre- and Post-natal Periods** | Mean (SD) | 25^th^ percentile | 50^th^ percentile | 75^th^ percentile | 95^th^ percentile |
| Pregnancy until delivery (9 months) | 0.11  (0.86) | 0 | 0 | 0 | 1 |
| Delivery to six months | 0.13  (1.04) | 0 | 0 | 0 | 1 |
| Six to 12 months post-delivery | 0.14  (0.82) | 0 | 0 | 0 | 1 |
| 12 to 24 months post-delivery | 0.31  (1.47) | 0 | 0 | 0 | 2 |
| 24 to 60 months post-delivery | 1.20  (4.27) | 0 | 0 | 1 | 7 |
| Delivery to 60 months | 1.78  (5.73) | 0 | 0 | 1 | 9 |
| **Pre- and Post-natal Periods** | **Cost for MH care ($)** | | | | |
| Pregnancy until delivery (9 months) | 5.51 (51.04) | 0 | 0 | 0 | 16.04 |
| Delivery to six months | 5.75 (51.96) | 0 | 0 | 0 | 29.57 |
| Six to 12 months post-delivery | 6.19 (43.10) | 0 | 0 | 0 | 31.08 |
| 12 to 24 months post-delivery | 13.76 (74.17) | 0 | 0 | 0 | 77.02 |
| 24 to 60 months post-delivery | 53.83 (212.64) | 0 | 0 | 15.10 | 279.96 |
| Delivery to 60 months | 72.52 (288.76) | 0 | 0 | 48.02 | 386.86 |
| **Pre- and Post-natal Periods** | **Cost of Prescription Drugs for MH ($)** | | | | |
| Pregnancy until delivery (9 months) | 3.19 (33.51) | 0 | 0 | 0 | 4.11 |
| Delivery to six months | 5.56 (30.84) | 0 | 0 | 0 | 18.23 |
| Six to 12 months post-delivery | 7.38 (40.50) | 0 | 0 | 0 | 26.62 |
| 12 to 24 months post-delivery | 18.46 (95.00) | 0 | 0 | 0 | 72.26 |
| 24 to 60 months post-delivery | 76.70 (473.28) | 0 | 0 | 10.62 | 338.20 |
| Delivery to 60 months | 108.10 (555.87) | 0 | 1.44 | 23.82 | 508.95 |

# **Table S4. Non-parametric regression discontinuity coefficients for number of visits, costs of mental health care and cost of prescription drug for mental health problems using the conventional method.**

| **Pre- and post-natal periods** | **Number of Visits for MH care** | **Cost for**  **MH care ($)** | **Cost of Prescription Drugs for MH ($)** |
| --- | --- | --- | --- |
| **Conventional method** | *B*  (SE) | *B*  (SE) | *B*  (SE) |
| Pregnancy until delivery (9 months) | -0.05 | -2.02 | 0.03 |
|  | (0.04) | (1.81) | (1.39) |
| Delivery to six months | -0.15 | -8.12 | 0.84 |
|  | (0.09) | (5.1) | (2.37) |
| Six to 12 months post-delivery | -0.12** | -4.61* | -4.24 |
|  | (0.04) | (1.92) | (3.3) |
| 12 to 24 months post-delivery | -0.08 | -3.33 | -6.59 |
|  | (0.06) | (2.48) | (8.14) |
| 24 to 60 months post-delivery | 0.19 | 13.41 | -45.97 |
|  | (0.19) | (9.81) | (46.16) |
| Delivery to 60 months | -0.09 | -1.35 | -60.04 |
|  | (0.22) | (11.39) | (50.74) |

*Notes:*

1. MH = mental health*; B* = regression coefficient; SE = Standard errors.
2. Statistical significance: *>5%; **>1% ;
3. Each column and sub-period are a different estimation.
4. Conventional refers to the method of Hahn et al. (2001).
5. Double optimal bandwidth was estimated using following Calonico, Cattaneo, Russell, and Titiunik (2016) computational method.

# **Table S5. Non-parametric regression discontinuity impacts for number of visits, costs of mental health care and cost of prescription drug for mental health problems, restrained sample near the discontinuity point.**

| **Pre- and post-natal periods** | **Number of Visits for MH care** | **Cost for**  **MH care ($)** | **Cost of Prescription Drugs for MH ($)** |
| --- | --- | --- | --- |
| **Conventional** | *B*  (SE) | *B*  (SE) | *B*  (SE) |
| Pregnancy until delivery (9 months) | -0.02 | -1.31 | 0.53 |
|  | (0.04) | (1.86) | (1.76) |
| Delivery to six months | -0.19 | -11.01 | -1.06 |
|  | (0.12) | (7.09) | (4.28) |
| Six to 12 months post-delivery | -0.09 * | -3.75* | -3.74 |
|  | (0.04) | (1.87) | (4.09) |
| 12 to 24 months post-delivery | -0.1 | -4.53 | -4.3 |
|  | (0.07) | (2.93) | (10.89) |
| 24 to 60 months post-delivery | 0.11 | 9.02 | -13.36 |
|  | (0.22) | (11.31) | (61.62) |
| Delivery to 60 months | -0.25 | -10.28 | -22.46 |
|  | (0.3) | (15.38) | (67.12) |

*Notes:*

1. Sample is restricted to mothers delivering 1 week prior (25-31 Dec. 2000) and after (1-7 Jan. 2007) the change in maternity leave policy.
2. N=1067 for number of visits and cost of mental health care
3. N=283 for cost of prescription drugs.
4. Because of the restricted sample when restraining the samples to mothers who delivered during the one week before and after the discontinuity point, we only model the conventional approach.
5. MH = mental health*; B* = regression coefficient; SE = Standard errors.
6. Statistical significance: *>5%; **>1% .

# **Table S6. Non-parametric regression discontinuity impacts for number of visits, costs of mental health care and cost of prescription drug for mental health problems, 99th percentile removed**

| **Pre- and post-natal periods** | **Number of Visits for MH care** | **Cost for**  **MH care ($)** | **Cost of Prescription Drugs for MH ($)** |
| --- | --- | --- | --- |
| **Conventional** | ***B* (SE)** | ***B* (SE)** | ***B* (SE)** |
| Pregnancy until delivery (9 months) | 0.01 | 0.57 | -0.32 |
|  | (0.01) | (0.52) | (0.37) |
| Delivery to six months | -0.01 | 0.19 | 0.28 |
|  | (0.02) | (0.64) | (1.01) |
| Six to 12 months post-delivery | -0.04* | -1.6* | -1.6 |
|  | (0.02) | (0.74) | (1.94) |
| 12 to 24 months post-delivery | -0.02 | -0.47 | -4.14 |
|  | (0.04) | (1.54) | (4.07) |
| 24 to 60 months post-delivery | -0.09 | -2.42 | -25.06 |
|  | (0.11) | (4.65) | (12.92) |
| Delivery to 60 months | -0.29 | -8.97 | -48.71* |
|  | (0.16) | (6.11) | (22.48) |
| **Bias-Corrected Robust** | ***B* (SE)** | ***B* (SE)** | ***B* (SE)** |
| Pregnancy until delivery (9 months) | 0.02 | 0.99 | -0.45 |
|  | (0.02) | (0.73) | (0.49) |
| Delivery to six months | 0.01 | 0.14 | 0.1 |
|  | (0.03) | (0.93) | (1.4) |
| Six to 12 months post-delivery | -0.07** | -2.28* | -4.23 |
|  | (0.02) | (1.09) | (3.04) |
| 12 to 24 months post-delivery | -0.03 | -2.11 | -12.97* |
|  | (0.05) | (2.15) | (6.28) |
| 24 to 60 months post-delivery | -0.2 | -4.8 | -46.52* |
|  | (0.16) | (6.73) | (19.28) |
| Delivery to 60 months | -0.44 | -9.54 | -80.23* |
|  | (0.23) | (9.12) | (35.16) |

*Notes:*

1. MH = mental health*; B* = regression coefficient; SE = Standard errors.
2. Statistical significance: *>5%; **>1%.
3. Each column and sub-period are a different estimation.
4. Conventional refers to the method of Hahn et al. (2001) bias-corrected robust refers to Calonico, Cattaneo, and Titiunik (2014).
5. Double optimal bandwidth was estimated using following Calonico, Cattaneo, Russell, and Titiunik (2016) computational method.

# **Table S7. Non-parametric regression discontinuity coefficients for number of visits, costs of mental health care and cost of prescription drug for mental health problems using the robust method and adjusting for type of delivery (cesarian vs vaginal).**

| **Pre- and post-natal periods** | **Number of Visits for MH care** | **Cost for**  **MH care ($)** | **Cost of Prescription Drugs for MH ($)** |
| --- | --- | --- | --- |
| **Conventional** | ***B* (SE)** | ***B* (SE)** | ***B* (SE)** |
| Pregnancy until delivery (9 months) | -0.03 | -1.89 | -0.03 |
|  | (0.03) | (1.86) | (1.39) |
| Delivery to six months | -0.14 | -5.59 | 0.79 |
|  | (0.09) | (3.60) | (2.41) |
| Six to 12 months post-delivery | -0.11* | -4.48* | -4.28 |
|  | (0.04) | (1.91) | (3.30) |
| 12 to 24 months post-delivery | -0.06 | -3.31 | -6.13 |
|  | (0.06) | (2.49) | (8.01) |
| 24 to 60 months post-delivery | 0.25 | 13.18 | -46.99 |
|  | (0.19) | (9.76) | (46.14) |
| Delivery to 60 months | -0.04 | -1.11 | -60.74 |
|  | (0.23) | (11.37) | (50.68) |
| **Bias-Corrected Robust** | ***B* (SE)** | ***B* (SE)** | ***B* (SE)** |
| Pregnancy until delivery (9 months) | -0.01 | - 0.97 | -2.06 |
|  | (0.05) | (2.82) | (1.76) |
| Delivery to six months | -0.21 | -9.31 | -1.99 |
|  | (0.14) | (6.36) | (3.95) |
| Six to 12 months post-delivery | -0.11* | -5.09* | -5.99 |
|  | (0.05) | (2.52) | (5.12) |
| 12 to 24 months post-delivery | -0.13 | -6.07 | -16.91 |
|  | (0.08) | (3.65) | (13.16) |
| 24 to 60 months post-delivery | 0.29 | 20.31 | -24.85 |
|  | (0.28) | (14.12) | (67.36) |
| Delivery to 60 months | -0.08 | -1.14 | -54.12 |
|  | (0.33) | (16.15) | (75.52) |

*Notes:*

1. MH = mental health*; B* = regression coefficient; SE = Standard errors.
2. Statistical significance: *>5%; **>1%.
3. Each column and sub-period are a different estimation.
4. Conventional refers to the method of Hahn et al. (2001) bias-corrected robust refers to Calonico, Cattaneo, and Titiunik (2014).
5. Double optimal bandwidth was estimated using following Calonico, Cattaneo, Russell, and Titiunik (2016) computational method.

*Notes:*

1. MH = mental health*; B* = regression coefficient; SE = Standard errors.
2. Statistical significance: *>5%; **>1% ;
3. Each column and sub-period are a different estimation.
4. Conventional refers to the method of Hahn et al. (2001).
5. Double optimal bandwidth was estimated using following Calonico, Cattaneo, Russell, and Titiunik (2016) computational method.

# **Figure S1. Birth pattern in the one month prior and after the change of maternity leave policy**

# **Figure S2. Running variable density test**


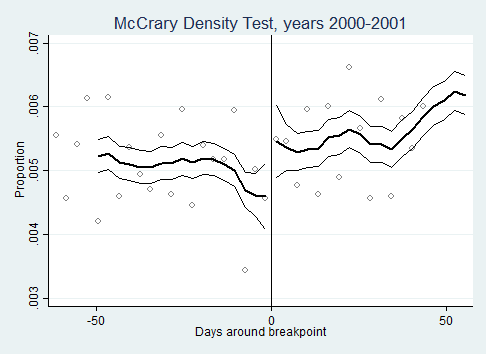


Note: McCrary density test results, 2000-2001. Bin size of 3, bandwidth of 11.

Standard errors presented on either side. Two months presented on each side of breakpoint.

# **Figure S3: RDD chart of mental health costs by periods around the discontinuity point.**


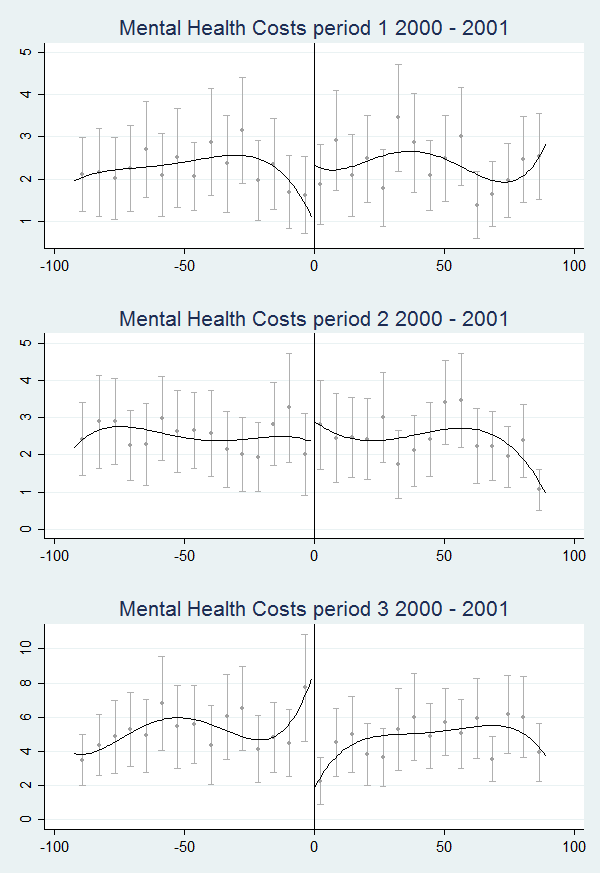

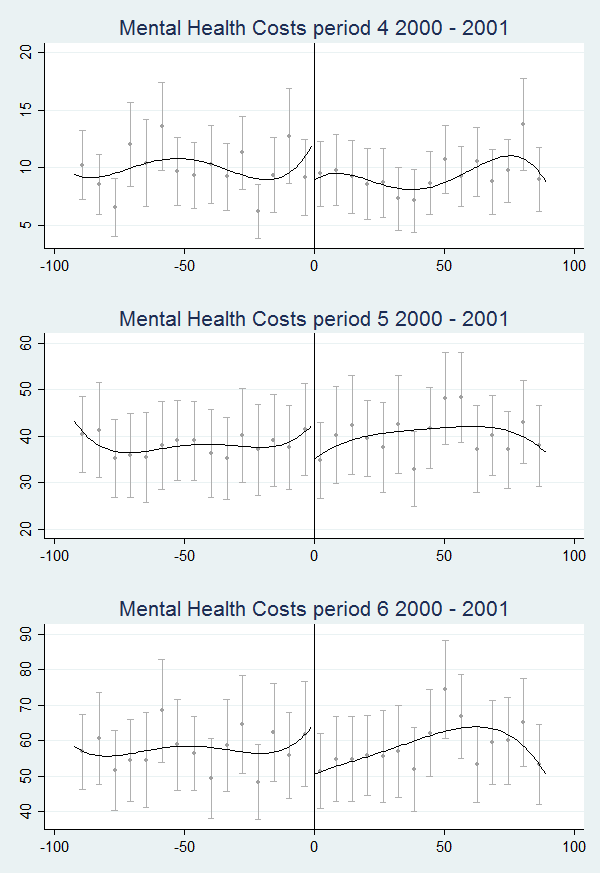


*Notes.*

1. Period 1 = Pregnancy until delivery (9 months); Period 2 = Delivery to six months; Period 3 = Six to 12 months post-delivery; Period 4: 12 to 24 months post-delivery; Period 5 = 24 to 60 months post-delivery; Period 6 = Delivery to 60 months.
2. The Y axis represents dollars in Canadian currency ($CAN).
3. The X axis represents the number of days since the discontinuity point.
4. Estimation markers are displayed with their 95% confidence intervals.
5. The coefficients are drawn from the analysis using the bias-corrected robust method by Calonico, Cattaneo, and Titiunik (2014).
6. Double optimal bandwidth was estimated using following Calonico, Cattaneo, Russell, and Titiunik (2016) computational method.
